# Microbiome analysis reveals the effects of black soldier fly oil on gut microbiota in pigeon

**DOI:** 10.3389/fmicb.2022.998524

**Published:** 2022-09-06

**Authors:** Suzhen Liu, Houqiang Luo, Meng Wang, Qingyan Wang, Longchuan Duan, Qingsong Han, Siwei Sun, Caixia Wei, Junjie Jin

**Affiliations:** Institute of Animal Husbandry and Veterinary Medicine and Institute of Pet Research, Wenzhou Vocational College of Science and Technology, Wenzhou, China

**Keywords:** black soldier fly oil, gut microbiota, 16S rDNA, pigeon, positive effect

## Abstract

The gut microbiota plays a vital roles in poultry physiology, immunity and metabolism. Black soldier fly oil is known to have a positive effect on the gut microbiota. However, the specific effect of black soldier fly oil on the composition and structure of the gut microbiota of the pigeon is unknown. In this experiment, 16S rDNA high-throughput sequencing was performed to study the effect of different doses of black soldier fly oil on the changes of pigeon intestinal microbes. Results indicated that the different doses of black soldier fly oil had no effect on the gut microbial diversity of the pigeon. Although the dominant phyla (*Proteobacteria*, *Firmicutes*, *Bacteroidetes*, and *Actinobacteria*) and genus (*uncultured_bacterium_f_Lachnospiraceae* and *Desulfovibrio*) in control group and experimental group with different doses were the same, the abundances of some beneficial bacteria (*Megasphaera*, *Intestinimonas*, *Prevotella_9*, *Lachnospiraceae_UCG-001*, *Faecalibacterium*, *Coprococcus_2*, *Parabacteroides*, *Megasphaera*, *Leuconostoc*, *Prevotellaceae_UCG-001*, *Lactococcus*, *Ruminococcaceae_UCG-014*, and *Coprococcus_2*) increased significantly as the concentration of black soldier fly oil increased. Taken together, this study indicated that black soldier fly oil supplementation could improve gut microbial composition and structure by increasing the proportions of beneficial bacteria. Notably, this is the first report on the effects of black soldier fly oil on the gut microbiota of pigeon, which contribute to understanding the positive effects of black soldier fly oil from the gut microbial perspective.

## Introduction

The poultry gastrointestinal tract is colonized with complex, diverse and changing microbial communities (Khan et al., 2020). Gut microbial communities play an important role in the health and productivity of the host (Li et al., 2021a). It is well known that in nutritional, metabolic, physiological and immune processes, the microbiota promotes the digestion and absorption of nutrients, stimulates host immune responses and improves resistance to disease (Lee et al., 2018; Bavananthasivam et al., 2021). These important gut microbial members include bacteria, fungi and protists, which together constitute and stabilize the complex intestinal microenvironment (Rychlik, 2020). Gastrointestinal microbes provide poultry with large amounts of enzymes, vitamins and proteins. Moreover, the microorganisms in the gastrointestinal tract of poultry can inhibit pathogenic bacteria through colonization resistance, immunomodulation and production of antibacterial substances such as hydrogen peroxide, bacteriocins and organic acids (Saleem et al., 2020; Wang et al., 2022). Studies have indicated that the imbalance of gut microbiota could cause a series of abnormal diseases in the body, such as chronic enteritis, liver disease and abdominal distension (Hu et al., 2021; Jian et al., 2021). Therefore, the effective stabilization of gut microbiota plays an important role in maintaining body health.

As one of the poultry, the squab refers to the young pigeons within 4 weeks of age, which has the advantages of rich nutrition and high medicinal value (Wen et al., 2022). With the large-scale breeding of pigeons, the diseases of pigeons has attracted increasing attention. Studies found that the gut microbiota disorder of pigeons can lead to a series of abnormal phenomena in the body. In order to alleviate the disturbance of gut microbiota, this study introduced black soldier fly oil as a dietary additive. According to reports, the crude fat content of black soldier fly larvae is 15–49%, and its body is rich in polyunsaturated fatty acids (Seyedalmoosavi et al., 2022). It contains a similar level of unsaturated fatty acids as fish oil and can be used as an additive in poultry diets (Liew et al., 2022). Studies have shown that black soldier fly oil can completely replace soybean oil in juvenile carp feed, and partially replace fish oil in rainbow trout feed (Mikolajczak et al., 2022). Therefore, black soldier fly oil is an effective alternative to dietary additives that needs to be further explored. Although it has been studied more in fish, its research in pigeons has rarely been introduced (Li et al., 2016).

With the development and application of new technologies such as macrogenomics and 16S rRNA gene sequencing, the sequencing and identification of gut microbiota have been well studied (Cuccato et al., 2021; Durazzi et al., 2021). High-throughput sequencing technology has been successfully applied to the analysis of gut microbial communities in dairy cows, goats and other animals, and has made important contributions to the diagnosis and treatment of various gastrointestinal diseases (Viale et al., 2017; Ma et al., 2018; Bhatia et al., 2020). We can better understand the composition and distribution of the gut microbiota of the pigeon through high-throughput sequencing of the gut microbiota of the pigeon. In this experiment, black soldier fly oil was added to the pigeon diet to explore its effect on the gut microbiota of pigeons, and combined with high-throughput sequencing technology to specifically analyze the role of black soldier fly oil in the adjustment of gut microbiota and other aspects. It can provide a theoretical basis for the production and application of black soldier fly oil in poultry and its mechanism of action. At the same time, it also provides a reference for the poultry production industry to seek economical, effective and environmentally friendly new feed additives.

## Materials and methods

### Sample acquisition

In this study, 80 28-day-old pigeons inoculated with Newcastle disease vaccine were randomly divided into 4 groups (control, 0.75, 1.5, and 3% black water gadfly oil supplementation groups), with 20 pigeons in each group. The formal experiment was started after 7 days of acclimation until the end of 65 days of age. During the experiment, all animals had free access to water. Record the feed consumption and the body weight in the early and late stage. Fecal samples obtained after the experiment were immediately stored in sterilized plastic tubes. After that, they were quickly frozen with liquid nitrogen and stored in a refrigerator at 80°C for further research.

### DNA extraction and illumine MiSeq sequencing

Bacterial genomic DNA was extracted based on the instructions of the QIAamp DNA Mini Kit (QIAGEN, Hilden, Germany). PCR amplification primers were designed to amplify the v3/v4 regions of the 16S rDNA gene (338F: ACTCCTACGGGAGGCAGCA and 806R: GGACTACHVGGGTWTCTAAT) using the diluted genomic DNA as a template. PCR amplification was repeated 3 times under the same conditions to ensure the accuracy of the experimental results. Genomic DNA was separated and quality assessed by 0.8% (w/v) agarose gel electrophoresis and quantified by UV-Vis spectrophotometer (NanoDrop 2000, United States). According to the molecular weight of 16S rDNA, a suitable size of gel was cut, and the 16S rDNA was recovered and purified according to the instructions of the gel recovery kit (Axygen, CA, United States). According to the preliminary electrophoresis results, the recovered and purified PCR products were subjected to fluorescence quantitative detection. Then the samples were mixed in the corresponding proportions according to the fluorescence quantification results. The purified PCR products were used to construct a sequencing library according to the specifications of Illumina TruSeq (Illumina, United States), and the quality detection and fluorescence quantification of the initial library were performed. A single peak and a library with a concentration of more than 2 nM were identified as qualified libraries, and the final qualified libraries were diluted and mixed in proportion. Finally, high-throughput sequencing was performed using the MiSeq sequencer.

### Bioinformatics and statistical analysis

Trimmomatic software was used to de-cluster the original paired-end sequence after sequencing, and the de-cluttered paired-end sequence was spliced with FLASH (v1.2.7) software. Chimera sequences in the removed sequences were detected using UCHIME (v4.2) software. After sequencing data preprocessing to generate high-quality sequences, VSEARCH (1.9.6) software was used to divide operational taxonomic units (OTUs) according to 97% similarity, and the most abundant sequences in each OTU were selected as representative sequences. Meanwhile, phylogenetic analysis and multiple sequence alignment of different OTUs were performed using MUSCLE software, with a confidence threshold of 0.8. Before diversity analysis, rarefaction curves were generated to assess the sequencing depth of each sample. According to the measured effective data, alpha diversity and beta diversity were analyzed successively. Multiple indicators such as Shannon, ACE, Chao1, and Good’s coverage were calculated, and the similarities and differences of the gut microbial communities of each group were observed. SPSS (v19.0) was used for statistical analysis of experimental data, and all data were expressed as mean ± SEM. *P*-value < 0.05 indicates statistical significance.

## Results

### Data acquisition and analysis

**FIGURE 1:**
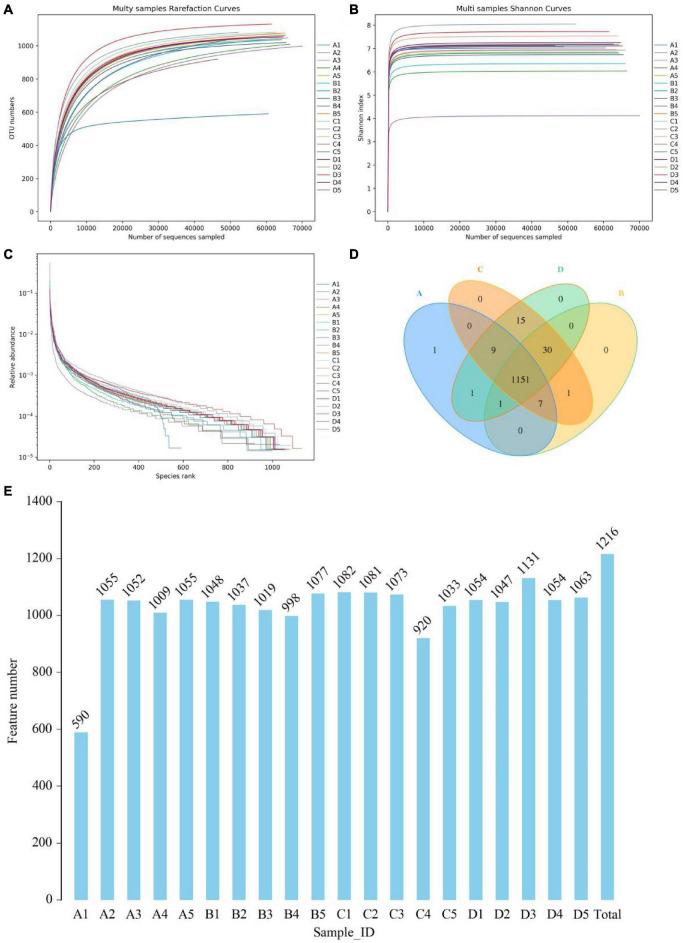
Analysis of samples feasibility and OTUs composition. Sequencing depth and evenness were reflected by rank abundance **(A,B)** and rarefaction curves **(C)**. Venn diagrams for OTUs composition and distribution **(D,E)**.

In this amplicon sequencing, a total of 400,159, 400,237, 400,454, and 400,633 raw sequences were collected from A, B, C and D groups, respectively (Table 1). After quality test, 1,527,293 high-quality reads (A = 382,402, B = 386,420, C = 372,770, D = 385,701) were acquired from 20 samples, with an average of 76,364 (ranging from 66,011 to 77,590) reads per sample. Results of rarefaction and rank abundance curves indicated that nearly all the microbial species could be recognized (Figures 1A–C). Based on 97% nucleotide-sequence similarity, 1216 OTUs (A = 1,170, B = 1,190, C = 1,213, D = 1,207) were totally identified, ranging from 590 to 1,131 OTUs per sample (Figures 1D,E).

**TABLE 1:** The sequence information of each sample.

Sample	Raw reads	Clean	Effective reads	AvgLen	GC	Q20	Q30	Effective
		reads		(bp)	(%)	(%)	(%)	(%)
A1	80,039	79,592	75,066	419	53.84	99.15	96.41	93.79
A2	80,119	79,709	76,902	415	54.74	99.16	96.49	95.98
A3	80,140	79,715	76,760	414	54.77	99.17	96.52	95.78
A4	80,008	79,556	76,898	413	55.14	99.18	96.56	96.11
A5	79,853	79,418	76,776	414	54.86	99.18	96.55	96.15
B1	79,991	79,559	77,140	414	54.96	99.17	96.54	96.44
B2	80,053	79,610	77,189	414	54.81	99.18	96.56	96.42
B3	79,806	79,371	77,078	414	54.8	99.19	96.57	96.58
B4	79,998	79593	77,423	423	53.52	99.15	96.39	96.78
B5	80,389	79,937	77,590	414	54.77	99.18	96.57	96.52
C1	80,167	79,767	77,427	414	54.83	99.2	96.62	96.58
C2	80,366	80,001	76,525	415	54.58	99.21	96.62	95.22
C3	80,220	79,811	77,089	416	54.46	99.19	96.56	96.1
C4	79,771	79,354	66,011	418	54.13	99.17	96.48	82.75
C5	79,930	79,510	75,718	415	53.96	99.19	96.57	94.73
D1	80,281	79,889	77,336	415	54.62	99.2	96.62	96.33
D2	80,174	79,702	77,248	415	54.78	99.14	96.38	96.35
D3	79,996	79,529	76,874	415	54.7	99.15	96.42	96.1
D4	79,819	79,374	76,901	415	54.63	99.16	96.45	96.34
D5	80,363	79,879	77,342	415	54.51	99.17	96.47	96.24

### Comparative analysis of gut microbial diversity

**FIGURE 2:**
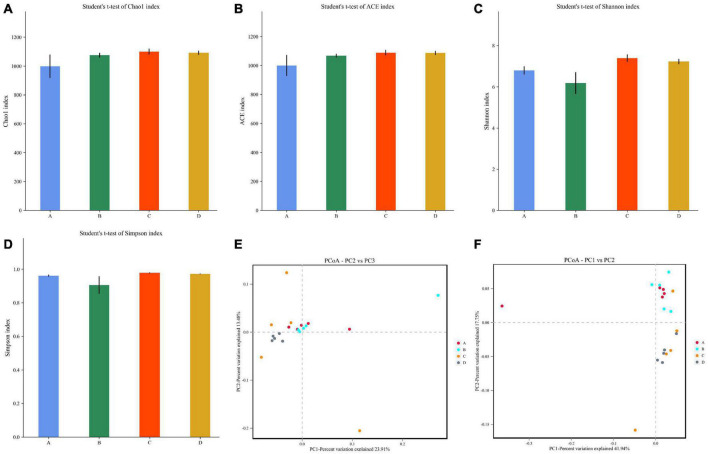
Comparative analysis of microbial diversity between control and black soldier fly oil treatment groups. **(A–D)** Represent Chao, ACE, Shannon and Simpson indices, respectively. **(E,F)** Represent scatterplot from PCoA based on weighted and unweighted UniFrac distances.

To further explore the effect of black soldier fly oil administration on gut microbiota of pigeon, we computed alpha and beta diversity indexes that could reflect gut microbial diversity. Good’s coverage estimates in the A, B, C, and D groups were almost 100%, showing excellent coverage. The average of Chao1 index in A, B, C, and D were 998.32, 1075.30, 1099.80, and 1091.99, while the ACE index was 999.79, 999.79, 1088.38, and 1086.71, respectively (Figures 2A,B). Furthermore, the average of Shannon index in A, B, C, and D groups were 6.80, 6.18, 7.39, and 7.23, while the Simpson index were 0.96, 0.90, 0.97, and 0.97, respectively (Figures 2C,D). Comparative analysis of microbial diversity indices showed that there was no significant difference in the Chao1, ACE, Simpson and Shannon indices between control and experimental groups regardless of the treatment dose. Alpha-diversity analysis revealed that black soldier fly oil administration had no obvious effect on the gut microbial diversity and abundance of pigeon. PCoA that reflects the gut microbial similarities and differences among different individuals was applied to dissect the gut microbial beta-diversity. Results of beta-diversity analysis indicated that the individuals in A, B, C, and D groups were clustered together, suggesting that black soldier fly oil administration had no effect on the major components of the gut microbiota (Figures 2E,F).

### Comparative analysis of microbial taxonomic composition

**FIGURE 3:**
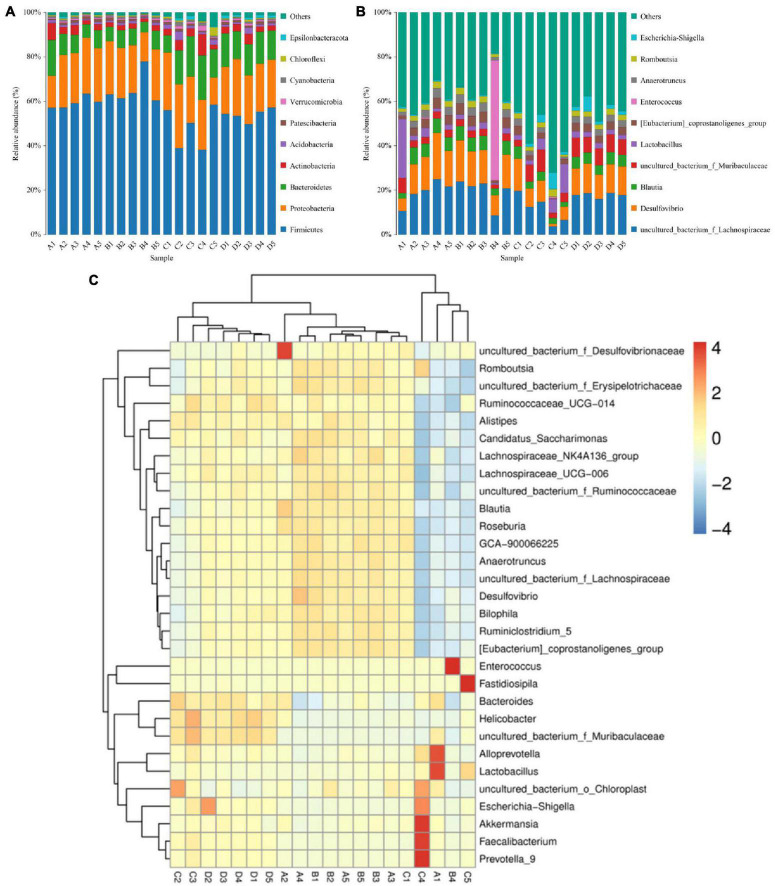
The percentages of dominant bacterial taxa at the level of phylum **(A)** and genus **(B)**. The color-block m the heatmap represents the normalized relative abundance of each bacterial **(C)** genera.

**FIGURE 4:**
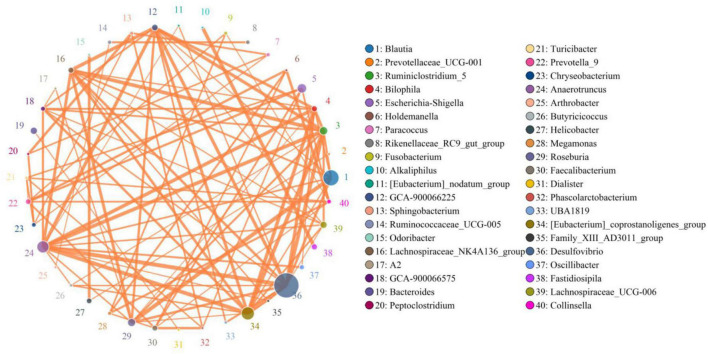
The correlations between different bacteria was assessed by network diagram. The orange lines indicate positive correlation.

In the present microbiome analysis, a total of 29 phyla and 481 genera were recognized in A, B, C, and D groups, ranging from 21 to 29 phyla and 271 to 454 genera per sample, respectively (Figures 3A,B). Specifically, the phyla *Firmicutes* (59.39, 65.50, 48.87, and 54.01%), *Proteobacteria* (22.06, 20.75, 22.25, and 22.42%), *Bacteroidetes* (9.56, 6.96, 14.53, and 13.81%) and *Actinobacteria* (3.86, 2.11, 4.38, and 3.11%) were the four most dominant phyla in the A, B, C, and D groups regardless of treatment, accounting for over 99.00% of the total composition. Other phyla such as *Cyanobacteria* (0.67, 0.63, 1.04, and 0.61%), *Verrucomicrobia* (0.60, 0.51, 1.11, and 0.71%), *Epsilonbacteraeota* (0.35, 0.23, 0.78, and 1.10%) and *Chloroflexi* (0.41, 0.37, 1.15, and 0.49%) in the A, B, C, and D groups were identified in lower abundances. At the genus level, *uncultured_bacterium_f_Lachnospiraceae* (19.25, 19.47, 12.14, and 17.87%) and *Desulfovibrio* (14.33, 14.65, 8.40, and 12.39%) were the most prevalent bacteria in the A, B, C, and D groups regardless of treatment. The distribution and correlation of predominant bacteria of A, B, C, and D groups could also be observed by the clustering heatmap and network diagram, respectively (Figures 3C, 4).

**FIGURE 5:**
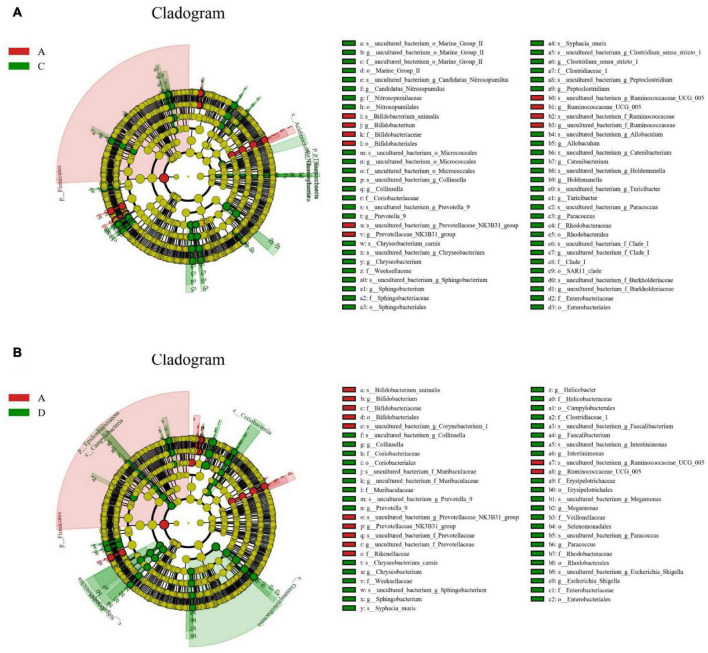
Identification of differential taxa associated with black soldier fly oil administration in pigeon. Each circle in cladogram represents a bacterial taxon and red and green circles indicate differential taxa. **(A,B)** Cladogram indicated the phylogenetic distribution of gut microbiota.

To further assess the influences of black soldier fly oil administration on the gut microbiota in pigeon, we conducted Metastats analysis on different classification levels (Tables 2– 4). At the phyla level, the relative abundances of *Spirochaetes*, *Thaumarchaeota*, *Euryarchaeota* in the C group and *Epsilonbacteraeota*, *Deinococcus-Thermus*, *Spirochaetes*, and *Bacteroidetes* in the D group were higher than those in the A group. At the genus level, *Wolbachia*, *Rothia*, *Ruminococcaceae_UCG-004*, *Ruminococcaceae_UCG-010*, *Veillonella*, *Blautia*, *Anaerotruncus*, *Ruminococcaceae_UCG-005*, *Anaeromyxobacter*, *Centipeda*, and *Faecalibaculum* were significantly more dominant in the A group than in the C group, whereas the *Clostridium_sensu_stricto_1*, *Candidatus_Actinomarina*, *Leifsonia*, *Megasphaera*, *Fluviicola*, *Leuconostoc*, *Allisonella*, *Synechococcus_CC9902*, *Candidatus_Nitrosopumilus*, *Lachnospiraceae_UCG-001*, *Terrimonas*, *NS5_marine_group*, and *Lawsonia* were lower. Moreover, a comparison of the D group and A group showed an obvious increase in the abundances of *Allobaculum*, *Sphingobacterium*, *Prevotella_9*, *Anaeroplasma*, *Peptoclostridium*, *Lachnospiraceae_UCG-001*, *Megamonas*, *Chryseobacterium*, *Faecalibacterium*, *Anaerobiospirillum*, *Paracoccus*, *Helicobacter*, *Phascolarctobacterium*, *Holdemanella*, *Serratia*, *Gelidibacter*, *Catenibacterium*, *Luteimonas*, *Collinsella*, *Muribaculum*, *Caldicoprobacter*, *Intestinimonas*, *Moheibacter*, *Slackia*, *Sutterella*, *Erysipelotrichaceae_UCG-003*, *Deinococcus*, *Turicibacter*, *Alkanindiges*, *Coprococcus*_2, *Parabacteroides*, *Aequorivita*, *Lawsonia*, *Megasphaera*, *Solobacterium*, *Leuconostoc*, *Angelakisella*, *Leifsonia*, *Lysobacter*, *Ketogulonicigenium*, *Tyzzerella_3*, *Paraburkholderia_tropica*, *Fusobacterium*, *Candidatus_Koribacter*, *Peptostreptococcus*, *Prevotellaceae_UCG-001*, *Odoribacter*, *Subdoligranulum*, *Allisonella*, *Asticcacaulis*, *Brevinema*, *Imperialibacter*, *Ruminococcaceae_UCG-014*, *Candidatus_Actinomarina*, *Candidatus_Aquiluna*, *OM60NOR5_clade*, *Synechococcus_CC9902*, *Clostridium_sensu_stricto_1*, *Lachnospiraceae_ND3007_group*, *Hafnia-Obesumbacterium*, *Lactococcus* and *Flavonifractor* as well as a distinct decrease in the abundances of *Rikenella*, *Rothia*, *Wolbachia* and *Ruminococcaceae_UCG-005*. Similar results were also observed in LEfSe analysis (Figure 5 and Supplementary Figure 1).

**TABLE 2:** Comparative analysis of differential bacteria between A and B groups.

Taxa	A (%)	B (%)	*P*
*Rikenella*	0.15 ± 0.012	0.10 ± 0.011	0.013
*Terrisporobacter*	0.0087 ± 0.0019	0.018 ± 0.0032	0.025
*Escherichia-Shigella*	0.71 ± 0.04	0.52 ± 0.070	0.03
*Plantibacter*	0.068 ± 0.017	0.029 ± 0.0056	0.039
*Rothia*	0.15 ± 0.033	0.075 ± 0.0077	0.04
*Candidatus_Arthromitus*	0.064 ± 0.023	0.014 ± 0.0042	0.044

A and B indicated the control and low dose black soldier fly oil treatment groups, respectively. Data were indicated as mean ± SD.

**TABLE 3:** Comparative analysis of differential bacteria between A and C groups.

Taxa	A (%)	C (%)	*P*
*Spirochaetes*	0.044 ± 0.018	0.22 ± 0.066	0.0082
*Firmicutes*	59.3 ± 1.16	48.4 ± 4.22	0.012
*Thaumarchaeota*	0.068 ± 0.020	0.34 ± 0.12	0.035
*Euryarchaeota*	0.058 ± 0.012	0.26 ± 0.097	0.035
*Wolbachia*	0.042 ± 0.0070	0.011 ± 0.0049	0.0026
*Rothia*	0.15 ± 0.032	0.031 ± 0.0070	0.0029
*Ruminococcaceae_UCG-004*	0.35 ± 0.023	0.18 ± 0.043	0.0032
*Clostridium_sensu_stricto_1*	0.095 ± 0.018	0.40 ± 0.11	0.012
*Candidatus_Actinomarina*	0.046 ± 0.0087	0.17 ± 0.047	0.013
*Leifsonia*	0.0046 ± 0.0014	0.021 ± 0.0066	0.016
*Ruminococcaceae_UCG-010*	0.012 ± 0.0037	0.0023 ± 0.0018	0.018
*Megasphaera*	0.0093 ± 0.0039	0.045 ± 0.014	0.02
*[Eubacterium]_xylanophilum_group*	0.058 ± 0.0087	0.032 ± 0.0066	0.02
*Fluviicola*	0.0034 ± 0.0010	0.022 ± 0.0082	0.028
*Leuconostoc*	0.031 ± 0.0091	0.058 ± 0.0076	0.028
*Allisonella*	0.0047 ± 0.0014	0.015 ± 0.0045	0.031
*Synechococcus_CC9902*	0.042 ± 0.014	0.11 ± 0.031	0.031
*Candidatus_Nitrosopumilus*	0.060 ± 0.017	0.30 ± 0.11	0.033
*Veillonella*	0.17 ± 0.041	0.065 ± 0.029	0.034
*Blautia*	5.71 ± 0.86	3.46 ± 0.633	0.037
*Anaerotruncus*	3.2 ± 0.45	1.74 ± 0.534	0.039
*Lachnospiraceae_UCG-001*	0 ± 0	0.014 ± 0.0069	0.04
*Terrimonas*	0.0074 ± 0.0026	0.019 ± 0.0054	0.041
*NS5_marine_group*	0.0015 ± 0.00099	0.025 ± 0.011	0.042
*Ruminococcaceae_UCG-005*	0.52 ± 0.13	0.23 ± 0.055	0.045
*HIMB11*	0.022 ± 0.010	0.13 ± 0.056	0.046
*Anaeromyxobacter*	0.051 ± 0.015	0.016 ± 0.0068	0.046
*Centipeda*	0.0081 ± 0.0036	0.0010 ± 0.00041	0.047
*Faecalibaculum*	0.22 ± 0.061	0.092 ± 0.024	0.049
*Lawsonia*	0.053 ± 0.0098	0.11 ± 0.030	0.049

A and C indicated the control and medium dose black soldier fly oil treatment groups, respectively. Data were indicated as mean ± SD.

**TABLE 4:** Comparative analysis of differential bacteria between A and D groups.

Taxa	A (%)	D (%)	*P*
*Epsilonbacteraeota*	0.35 ± 0.11	1.1 ± 0.096	0.0004
*Deinococcus-Thermus*	0.0089 ± 0.0089	0.068 ± 0.012	0.0028
*Firmicutes*	59.3 ± 1.16	54 ± 1.23	0.0072
*Spirochaetes*	0.043 ± 0.018	0.10 ± 0.0090	0.011
*Bacteroidetes*	9.66 ± 1.73	13.8 ± 0.49	0.03
*Allobaculum*	0.0053 ± 0.0016	0.11 ± 0.0073	0.000016
*Sphingobacterium*	0.0012 ± 0.00058	0.21 ± 0.020	0.00005
*Prevotella_9*	0.22 ± 0.029	0.60 ± 0.024	0.000064
*Anaeroplasma*	0.0078 ± 0.0049	0.092 ± 0.0068	0.000079
*Peptoclostridium*	0.00031 ± 0.00031	0.14 ± 0.015	0.000093
*Lachnospiraceae_UCG-001*	0 ± 0	0.018 ± 0.0022	0.00012
*Megamonas*	0.080 ± 0.02	0.35 ± 0.029	0.00013
*Chryseobacterium*	0.025 ± 0.012	0.41 ± 0.053	0.00015
*Faecalibacterium*	0.26 ± 0.058	0.71 ± 0.031	0.00016
*Anaerobiospirillum*	0 ± 0	0.013 ± 0.0021	0.00023
*Paracoccus*	0.016 ± 0.0062	0.31 ± 0.045	0.00024
*Helicobacter*	0.29 ± 0.091	1.06 ± 0.092	0.00032
*Phascolarctobacterium*	0.079 ± 0.010	0.16 ± 0.011	0.0005
*Holdemanella*	0.019 ± 0.014	0.12 ± 0.013	0.00057
*Serratia*	0.047 ± 0.0098	0.14 ± 0.016	0.0006
*Gelidibacter*	0.0009 ± 0.00063	0.036 ± 0.0068	0.00064
*Catenibacterium*	0.012 ± 0.0015	0.12 ± 0.022	0.0007
*Luteimonas*	0.0024 ± 0.0010	0.020 ± 0.0037	0.0011
*Collinsella*	0.075 ± 0.032	0.34 ± 0.048	0.0011
*Muribaculum*	0.034 ± 0.015	0.13 ± 0.015	0.0011
*Caldicoprobacter*	0.0072 ± 0.0028	0.033 ± 0.0053	0.0013
*Intestinimonas*	0.20 ± 0.032	0.41 ± 0.036	0.0016
*Moheibacter*	0 ± 0	0.013 ± 0.0032	0.0024
*Slackia*	0.00031 ± 0.00031	0.038 ± 0.0097	0.0027
*Sutterella*	0.025 ± 0.0069	0.061 ± 0.0063	0.003
*Erysipelotrichaceae_UCG-003*	0.013 ± 0.0050	0.050 ± 0.0083	0.0032
*Deinococcus*	0.0089 ± 0.0089	0.068 ± 0.012	0.0033
*Turicibacter*	0.11 ± 0.017	0.20 ± 0.016	0.0033
*Alkanindiges*	0.012 ± 0.0077	0.065 ± 0.011	0.0035
*Coprococcus_2*	0.0065 ± 0.0021	0.025 ± 0.0045	0.0035
*Parabacteroides*	0.23 ± 0.013	0.32 ± 0.022	0.0037
*Aequorivita*	0 ± 0	0.011 ± 0.0031	0.0045
*Lawsonia*	0.053 ± 0.0098	0.095 ± 0.0068	0.005
*Megasphaera*	0.0093 ± 0.0039	0.028 ± 0.0039	0.0063
*Solobacterium*	0 ± 0	0.01 ± 0.0029	0.0063
*Rikenella*	0.14 ± 0.011	0.10 ± 0.0076	0.0066
*Leuconostoc*	0.031 ± 0.0091	0.071 ± 0.0079	0.0073
*Angelakisella*	0.028 ± 0.013	0.076 ± 0.0059	0.0073
*Leifsonia*	0.0046 ± 0.0014	0.013 ± 0.0022	0.0082
*Lysobacter*	0 ± 0	0.0093 ± 0.0029	0.0086
*Ketogulonicigenium*	0.0018 ± 0.0011	0.013 ± 0.0033	0.0089
*Tyzzerella_3*	0 ± 0	0.016 ± 0.0052	0.0091
*Paraburkholderia_tropica*	0 ± 0	0.020 ± 0.0068	0.0098
*Fusobacterium*	0.15 ± 0.031	0.29 ± 0.029	0.0099
*Candidatus_Koribacter*	0.0021 ± 0.0013	0.013 ± 0.0036	0.011
*Peptostreptococcus*	0.010 ± 0.0036	0.030 ± 0.0055	0.012
*Prevotellaceae_UCG-001*	0.10 ± 0.017	0.18 ± 0.02	0.012
*Hydrogenophaga*	0.015 ± 0.0044	0.0025 ± 0.00095	0.012
*Odoribacter*	0.11 ± 0.042	0.28 ± 0.041	0.012
*Subdoligranulum*	0.047 ± 0.0064	0.079 ± 0.0089	0.012
*Allisonella*	0.0047 ± 0.0014	0.013 ± 0.0025	0.013
*Asticcacaulis*	0.00094 ± 0.00062	0.0056 ± 0.0015	0.014
*Brevinema*	0.041 ± 0.016	0.094 ± 0.0091	0.014
*Imperialibacter*	0.012 ± 0.0053	0.035 ± 0.0067	0.017
*Ruminococcaceae_UCG-014*	0.71 ± 0.065	0.90 ± 0.037	0.021
*Rothia*	0.15 ± 0.032	0.061 ± 0.010	0.021
*Wolbachia*	0.042 ± 0.0070	0.019 ± 0.0053	0.022
*Candidatus_Actinomarina*	0.046 ± 0.0087	0.080 ± 0.010	0.025
*Candidatus_Aquiluna*	0.0062 ± 0.0026	0.018 ± 0.0043	0.025
*OM60NOR5_clade*	0.0034 ± 0.0013	0.021 ± 0.0071	0.026
*Synechococcus_CC9902*	0.042 ± 0.014	0.098 ± 0.017	0.028
*Clostridium_sensu_stricto_1*	0.095 ± 0.018	0.16 ± 0.022	0.03
*Lachnospiraceae_ND3007_group*	0.0044 ± 0.0022	0.012 ± 0.0028	0.035
*Hafnia-Obesumbacterium*	0.15 ± 0.036	0.27 ± 0.040	0.036
*Lactococcus*	0.061 ± 0.014	0.10 ± 0.011	0.039
*Flavonifractor*	0.0084 ± 0.0022	0.017 ± 0.0036	0.044
*Ruminococcaceae_UCG-005*	0.52 ± 0.13	0.21 ± 0.043	0.045

A and D indicated the control and high dose black soldier fly oil treatment groups, respectively. Data were indicated as mean ± SD.

## Discussion

Numerous studies indicated that gut microbiota is closely related to intestinal digestion and absorption, host metabolism and immune system maturation, which in turn depends on the normal and stable gut microbial composition (Khudhair et al., 2020; Overstreet et al., 2020). Thus, the investigation and analysis of gut microbial characteristics are of great significance to ensure the health of animals. As a crucial and sensitive indicator, gut microbiota is inevitably affected by the diet and external environment (Li et al., 2021b,2022a). Although growing evidence indicated the importance of black soldier fly oil in animal husbandry, but little is known about its influence on gut microbiota in pigeon. In this study, we first investigated the effects of black soldier fly oil administration on gut microbiota in pigeon and found significant improvements in gut microbiota.

Early surveys showed that gut microbial diversity and abundance are constantly changing under the influence of external factors including species, diet, age and even weather (Hu et al., 2016; Del et al., 2022). Generally, physiological fluctuations caused by these mild stimulation does not significantly alter intestinal function and homeostasis (Li et al., 2021c). However, intense stimulations such as organic contaminant, heavy metal and antibiotics may significantly decrease gut microbial diversity, causing gut microbial dysbiosis (Forouzandeh et al., 2021; Yang et al., 2021; Liao et al., 2022). Previous studies indicated that gut microbial dysbiosis not only cause multiple gastrointestinal diseases but also impair liver and brain causing systemic effect (Ya et al., 2020; Wan et al., 2022). Moreover, gut microbial dysbiosis may also be a core or driver factor of multiple diseases such as diabetes and inflammatory bowel diseases (Lin et al., 2021). Presently, gut microbial diversity is considered as an important indicator for assessing intestinal homeostasis (Sitarik et al., 2020; Zhang et al., 2022). In this study, we observed that black soldier fly oil supplementation did not alter gut microbial diversity and abundance of pigeon, demonstrating no effect on intestinal homeostasis. Scatterplot from PCoA revealed that the all the samples are clustered together, indicating that black soldier fly oil administration had no effect on the main components of the gut microbiota.

This study indicated that *Firmicutes*, *Bacteroidetes*, and *Proteobacteria* were abundantly present in all the samples regardless of the treatment. Notably, these phyla were also demonstrated to be dominant in the gut microbiota of chicken, duck and goose, showing their importance in intestinal ecosystem of poultry (Thiam et al., 2022; Yu et al., 2022). Interestingly, although the species of the dominant phyla did not change, the ratios of them changed significantly. At the phylum level, the proportions of *Epsilonbacteraeota*, *Deinococcus-Thermus*, *Spirochaetes*, and *Bacteroidetes* in the gut microbiota of BSFO-treated pigeon dramatically increased compared with control pigeon. *Bacteroidetes* participated in the positive regulation of the intestinal immune system and associated with the digestion of proteins and carbohydrates (Zhang et al., 2020). Importantly, black soldier fly oil administration also resulted in significant changes in the taxonomic composition of bacteria and these altered functional bacteria may play key roles in intestinal homeostasis and function. Interestingly, most of the changed bacteria including *Megasphaera*, *Intestinimonas*, *Prevotella_9*, *Lachnospiraceae_UCG-001*, *Faecalibacterium*, *Coprococcus_2*, *Parabacteroides*, *Megasphaera*, *Leuconostoc*, *Prevotellaceae_UCG-001*, *Lactococcus*, *Ruminococcaceae_UCG-014*, and *Coprococcus_2* display an upward trend, indicating that the present intestinal environment is more conducive to the survival of these bacteria and the improvement of the intestinal environment.

*Prevotella* has been demonstrated to participate in the carbohydrate metabolism (Downes et al., 2013). *Lachnospiraceae* was previously demonstrated to negatively correlated with intestinal inflammation (Zhao et al., 2017). As vital important intestinal symbiotic bacteria, *Faecalibacterium* and *Intestinimonas* can produce butyrate and possess the immunomodulatory and anti-inflammatory functions (Bui et al., 2016; Zhou et al., 2018). Butyrate is known to be able to decrease diabetes and angiocardiopathy caused by obesity (Wu et al., 2018; Noureldein et al., 2020). Additionally, the relative abundance of *Faecalibacterium* was dramatically decreased in colitis patient, showing its great potential in treating inflammatory bowel disease (Qiu et al., 2013). Previous research demonstrated that the relative abundance of *Parabacteroides* in the intestine is negatively closely related to obesity and diabetes, showing the vital roles in lipid and glucose metabolism (Wang et al., 2019). *Prevotellaceae* is responsible for carbohydrate food digestion, which may contribute to more energy capture for host (Li et al., 2021a). *Ruminococcaceae*, a potentially beneficial gut bacteria, contributes to improving immune function and intestinal environment of host (Gu et al., 2022). Moreover, *Ruminococcaceae* has also been demonstrate to decrease intestinal permeability and relieve liver cirrhosis (Xi et al., 2021). *Lactococcus* exhibit multiple beneficial biological characteristics such as promoting growth performance, enhancing immunity and maintaining gut microbial balance (Kakade et al., 2022; Werum et al., 2022). Moreover, *Lactococcus* also shows the capacity of secreting antimicrobial peptides and organic acids, indicating its key roles in maintaining intestinal homeostasis and inhibiting pathogenic bacteria (Feng et al., 2019; Xia et al., 2019). Notably, some increased bacterial genus such as *Coprococcus* and *Megasphaera* in experimental group are potential producers of short-chain fatty acids (SCFAs) (Yoshikawa et al., 2018; Ye et al., 2021). Earlier studies indicated that SCFAs was beneficial to maintain intestinal homeostasis and improve intestinal environment (Akhtar et al., 2022; Li et al., 2022b). Furthermore, SCFAs have also involved in the positive regulation of the cell proliferation, immunologic function and host metabolism (Lu et al., 2021; Li et al., 2022c). Importantly, the higher concentrations of SCFAs could decrease intestinal pH, which in turn inhibit the pathogen growth (He et al., 2019). *Leuconostoc* has long been regarded as potential probiotic in the intestine, which could secrete exopolysaccharide and regulate host immunologic function (Sun et al., 2020). Recent research on *Leuconostoc* has also revealed its antibacterial activities and great potential for relieving obese in mice caused by high-fat diets (Pan et al., 2022).

Increasing evidence indicated that animal gut microbiota is a complex dynamic system involving trillions of microorganisms (Ding et al., 2020; Liu et al., 2020). These gut-residing microorganisms could maintain intestinal homeostasis through interacting in the parasitic or symbiotic relationship (Knezevic et al., 2020; Xia et al., 2020). The maintenance of intestinal homeostasis is the premise for host to conduct complex intestinal function, whereas gut microbial dysbiosis may result in gut microbial dysbiosis and gastrointestinal and even systemic diseases (Iatcu et al., 2021; Liao et al., 2021). In this study, we also observed that some of the changed bacteria showed strong correlations. Therefore, these altered bacteria may affect the functions of others, which in turn amplifies the effect on intestinal function. Our results also conveyed an important message that black soldier fly oil administration not only directly improve gut microbial composition and structure of pigeon but also indirectly affected other functional bacteria via bacterial interaction, which may further maintain intestinal homeostasis and improve gut microbiota.

## Conclusion

Taken together, this study first dissected the influences of black soldier fly oil on gut microbiota of pigeon. Results demonstrated that black soldier fly oil administration, especially high doses, could alter gut microbial composition and structure, characterized by increased proportion of beneficial bacteria. Importantly, this study contributed to raising the public awareness of black soldier fly oil and conveying a vital message that gut microbial improvement may be one of the key pathways for black soldier fly oil to exert its beneficial effect.

## Data availability statement

The datasets presented in this study can be found in online repositories. The names of the repository/repositories and accession number(s) can be found below: https://www.ncbi.nlm.nih.gov/, PRJNA859657.

## Ethics statement

The animal study was reviewed and approved by the Wenzhou Vocational College of Science and Technology’s Animal Care Committee.

## Author contributions

SL and HL: research idea and methodology. HL, MW, QW, and LD: reagents, materials, and analysis tools. QH, SS, CW, and JJ: assist in pigeon experiments. HL: visualization. All authors approved the final manuscript.
