# Supplementary figures and images for: Microbiome analysis reveals the effects of black soldier fly oil on gut microbiota in pigeon
Source: Front Microbiol. 2022 Sep 6;13:998524. doi: 10.3389/fmicb.2022.998524 (PMC9495606; doi:10.3389/fmicb.2022.998524)

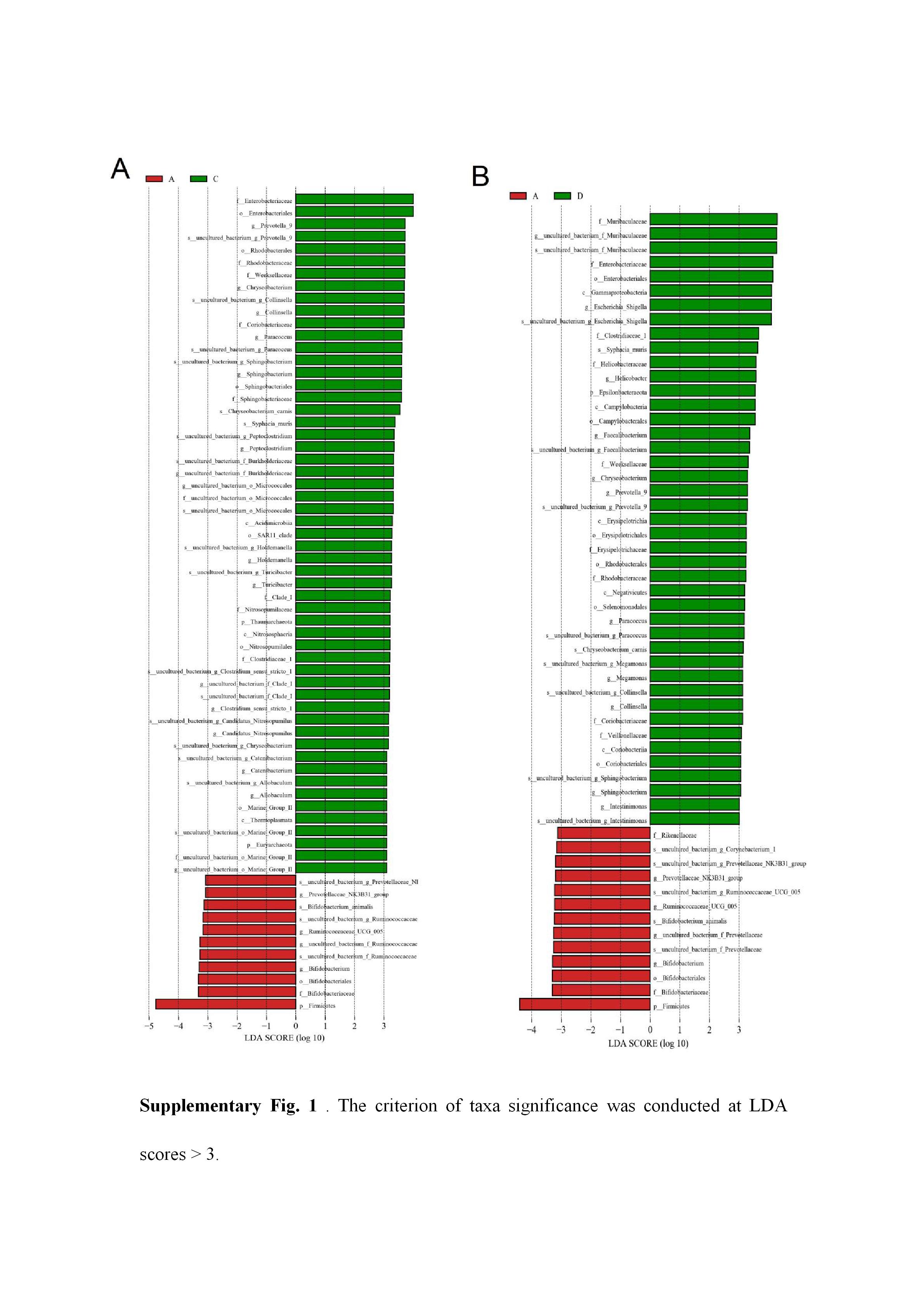

Supplement: Supplementary file 1 [file Image_1.jpeg]
